# Transport of thyroid hormones via the choroid plexus into the brain: the roles of transthyretin and thyroid hormone transmembrane transporters

**DOI:** 10.3389/fnins.2015.00066

**Published:** 2015-03-03

**Authors:** Samantha J. Richardson, Roshen C. Wijayagunaratne, Damian G. D'Souza, Veerle M. Darras, Stijn L. J. Van Herck

**Affiliations:** ^1^School of Medical Sciences, RMIT UniversityBundoora, VIC, Australia; ^2^Laboratory of Comparative Endocrinology, Biology Department, KU LeuvenLeuven, Belgium

**Keywords:** blood-cerebrospinal fluid barrier, brain, choroid plexus, development, evolution, thyroid hormones, transthyretin, thyroid hormone transporters

## Abstract

Thyroid hormones are key players in regulating brain development. Thus, transfer of appropriate quantities of thyroid hormones from the blood into the brain at specific stages of development is critical. The choroid plexus forms the blood-cerebrospinal fluid barrier. In reptiles, birds and mammals, the main protein synthesized and secreted by the choroid plexus is a thyroid hormone distributor protein: transthyretin. This transthyretin is secreted into the cerebrospinal fluid and moves thyroid hormones from the blood into the cerebrospinal fluid. Maximal transthyretin synthesis in the choroid plexus occurs just prior to the period of rapid brain growth, suggesting that choroid plexus-derived transthyretin moves thyroid hormones from blood into cerebrospinal fluid just prior to when thyroid hormones are required for rapid brain growth. The structure of transthyretin has been highly conserved, implying strong selection pressure and an important function. In mammals, transthyretin binds T4 (precursor form of thyroid hormone) with higher affinity than T3 (active form of thyroid hormone). In all other vertebrates, transthyretin binds T3 with higher affinity than T4. As mammals are the exception, we should not base our thinking about the role of transthyretin in the choroid plexus solely on mammalian data. Thyroid hormone transmembrane transporters are involved in moving thyroid hormones into and out of cells and have been identified in many tissues, including the choroid plexus. Thyroid hormones enter the choroid plexus via thyroid hormone transmembrane transporters and leave the choroid plexus to enter the cerebrospinal fluid via either thyroid hormone transmembrane transporters or via choroid plexus-derived transthyretin secreted into the cerebrospinal fluid. The quantitative contribution of each route during development remains to be elucidated. This is part of a review series on ontogeny and phylogeny of brain barrier mechanisms.

## Thyroid hormones

Thyroid hormones (THs) are key players in regulating development of the brain. Insufficient THs during prenatal development in humans can lead to cretinism and mental retardation, whereas insufficient THs in adult life can result in fatigue, lethargy, mental impairment, weight gain, cold intolerance and in severe cases, clinical depression. THs act mainly by regulating transcription of specific genes. THs are synthesized in the thyroid gland and are secreted into the blood. Most of the TH secreted from the thyroid gland is the precursor form (T4). About 80% of the active form (T3) is generated by deiodination of T4 to T3 in target tissues. In the blood, >99% of TH is bound to specific plasma proteins known as TH distributor proteins. Because THs are lipophilic they partition between the lipid phase and the aqueous phase with a ratio of 20,000:1 (Dickson et al., 1987). The binding of THs to TH distributor proteins prevents avid partitioning of THs into the lipid membranes of cells and ensures that there is a sufficiently large pool of TH circulating in the blood (Mendel et al., 1987). In humans, the main TH distributor proteins are albumin, transthyretin (TTR) and thyroxine-binding globulin (TBG), all of which are synthesized by the liver. In addition, a small fraction is distributed by lipoproteins including ApoB100 (Benvenga et al., 1989). The free hormone hypothesis states that it is the free hormone in blood (not the protein-bound hormone) that is important for biological activity (Mendel, 1989). Thus, THs must dissociate from the distributor proteins before they can exert their effects.

In human blood 99.97% of T4 and 99.70% of T3 is bound to the TH distributor proteins (Mendel, 1989). Of these, TBG has the highest affinity for T4 and T3 (1.0 × 10^10^ M^−1^ and 4.6 × 10^8^ M^−1^, respectively), TTR has intermediate affinity (7.0 × 10^7^ M^−1^ and 1.4 × 10^7^ M^−1^, respectively) and albumin has the lowest affinity (7.0 × 10^5^ M^−1^ and 1.0 × 10^5^ M^−1^, respectively). Together, these three TH distributor proteins form a buffering network for free T4 in blood, which could help to protect against hypothyroidism (abnormally low levels of free TH in blood) or hyperthyroidism (abnormally high levels of free TH in blood) (Schreiber and Richardson, 1997). The older literature and some modern text books state that the role of TH distributor proteins in blood is to overcome low TH solubility. However, the concentration of free T4 in human blood is about 25 pM and the maximum solubility of T4 in physiological saline is 2.3 μM, i.e., about 100,000 times the concentration of free T4 (Schreiber and Richardson, 1997). Therefore, TH distributor proteins must play a role other than to aid the solubility of THs in the blood and CSF.

The concentrations of each of the TH distributor proteins in adult human blood varies greatly: TBG at 0.015 g/l, TTR at 0.25 g/l and albumin at 42 g/l. Traditionally it has been believed that in blood, TBG distributes about 75% of thyroid hormones, TTR distributes 15% and albumin transports about 10%. Because TBG binds about 75%, it is sometimes referred to as the “most important” thyroid hormone distributor. However, this is too simplistic, as the biological importance is related to the delivery of THs to cells, which is dependent on the dissociation rates of T3 and T4 from the distributor proteins and capillary transit times. To determine which of the three TH distributor proteins contributes most effectively to hormone delivery to tissues, the dissociation rates and the capillary transit times have to be considered. In brief, the dissociation rates for T4 and T3 from TBG are 0.018 and 0.16 s^−1^, respectively; from TTR are 0.094 and 0.69 s^−1^, respectively and from albumin are 1.3 and 2.2 s^−1^, respectively (Mendel and Weisiger, 1990). Thus, given the capillary transit times for various tissues (Mendel et al., 1989), TTR is responsible for much of the immediate delivery of THs to tissues (Robbins, 2000).

THs have multiple mechanisms of action which can be grouped into “genomic” and “non-genomic” mechanisms of action. Non-genomic mechanisms include membrane-initiated mechanisms via binding to specific integrins (Davis et al., 2005, 2011; Lin et al., 2012); cytosolic kinase-initiated interactions which have non-genomic effects on glucose metabolism (Moeller et al., 2006, 2011) and rapid activation of kinases in signaling cascades in mitochondria resulting in altered fatty acid metabolism (Sayre and Lechleiter, 2012). The genomic pathway, which is generally better known and studied, involves thyroid hormone receptors (TRs). Free (unbound) THs enter target cells via the TH transmembrane transporters (Hennemann et al., 2001), which are responsible for influx and efflux of THs from cells. For genomic actions of THs within the cell nucleus, T4 can be deiodinated to T3 (considered the “active” form of TH). The family of deiodinases can either activate (e.g., T4 to T3) or inactivate (e.g., T3 to T2; T4 to rT3) THs within the cell (Darras et al., 2015). T3 exerts genomic effects by binding to TRs which are nuclear transcription factors. T3 can bind TRs either in the cytoplasm and translocate into the nucleus, or bind TRs in the nucleus. T3 binding to TR hetero-dimer complexes on thyroid response elements in the promotor of specific genes causes dissociation of co-repressor proteins and association of co-activator proteins. The resulting complex then initiates transcription of genes that are positively regulated by THs. Some genes are negatively regulated by THs, whereby binding of T3 induces the opposite changes and turns off transcription. Thus, it is crucial that the appropriate concentration of THs is present in the critical regions of the brain at each stage of development, to ensure normal brain maturation. This requires that sufficient (but not excess) THs move from the blood into the brain at critical stages of development. There are several pathways for THs to move from the blood into the CSF, but this review will focus on the route via the choroid plexus i.e., the blood-CSF barrier.

## The choroid plexus

The choroid plexus is a villous structure located in the lateral, third and fourth ventricles of the brain and is the site of the blood-CSF barrier. The structure of the choroid plexus is similar in all vertebrates studied to date (Ek et al., 2005). The blood supply to the choroid plexus comes from two posterior choroidal arteries, which branch from the internal carotid arteries. The rate of blood flow to the choroid plexus has been estimated to be about 3.5 faster than that to the rest of the brain (Zheng, 2005). The choroid plexus epithelial cells are joined by tight junctions, giving the tissue its barrier properties (Ek et al., 2005). The choroid plexus produces about 70% of the CSF, and thus has a major influence on its composition (Cserr, 1971). In humans the total CSF is replaced about four times per day (Brown et al., 2005). In comparison to the blood, which mixes, the CSF has a pipeline type of flow which does not mix. Consequently, the composition of the CSF can vary depending on the site from which it is sampled (Cserr, 1971). The choroid plexus barrier ensures that the composition of the CSF is very different to that of the blood. For example, in adult mammals, the CSF has only about 0.5% total protein concentration compared to that in the blood (Brown et al., 2005) and the relative ratios of proteins in the blood compared to the CSF differ markedly. However, the total protein concentration in the CSF changes during development, peaking early in development and then gradually decreasing to adult levels (Saunders, 1992). For a comprehensive summary of the development of the choroid plexus (see Ek et al., 2005).

## TTR synthesis by the choroid plexus

The first description of TTR synthesis in the brain was in 1985 (Soprano et al., 1985). Soon after, Schreiber's group identified TTR mRNA as being located only in the choroid plexus of the brain (Dickson et al., 1985) and then specifically in the choroid plexus epithelial cells (Stauder et al., 1986). The first experiments suggesting that TTR synthesis by the choroid plexus was involved in the movement of TH from the blood into the CSF were published in 1987. ^125^I-T4 was injected into the blood of rats and was detected in various brain structures: initially it accumulated in the choroid plexus to ~250% the level in blood. Thereafter, ^125^I-T4 accumulated in the CSF, after which it began to appear in the cortex and striatum of the brain. However, when the analogous experiment was repeated using ^125^I-T3, there was no specific accumulation of ^125^I-T3 in the choroid plexus. This was interpreted as TTR synthesis by the choroid plexus being involved in the movement of T4 but not T3, from the blood across the choroid plexus into the CSF (Dickson et al., 1987). TTR synthesized by the choroid plexus was shown to be secreted into the CSF and not into the blood (Schreiber et al., 1990; Duan et al., 1991) i.e., unidirectional secretion of TTR by the choroid plexus 100% into the CSF. Then Köhrle's group demonstrated that the TTR-T4 complex from the blood is not transported as such into the CSF; and the movement of T4 from the blood into the CSF is dependent on the concentration of free T4 in the serum and on T4 binding to choroid plexus-derived TTR i.e., if the TH binding capacity of TTR was disrupted, then TH did not cross the choroid plexus (Chanoine et al., 1992). Schreiber's group showed that if protein synthesis was blocked in a choroid plexus epithelial monolayer cell culture system, then T4 did not accumulate in the apical chamber (Southwell et al., 1993). Furthermore, the concentration of free T4 is higher in CSF than in the blood, thus, the unidirectional secretion of TTR (or TTR-T4) was moving T4 against the free T4 gradient (Southwell et al., 1993). These were the key experiments suggesting that the synthesis and secretion of TTR by the choroid plexus was involved in movement of T4 from the blood into the CSF.

The choroid plexus is the tissue with the highest concentration of TTR mRNA per gram tissue weight. The adult rat choroid plexus has an 11 times higher concentration of TTR mRNA compared to the liver (4.4 vs. 0.39 μg TTR mRNA per gram wet weight) (Schreiber et al., 1990). In chickens, the adult choroid plexus has a 22 times higher concentration of TTR mRNA compared to the liver (7.2 vs. 0.33 μg TTR mRNA per gram wet) (Duan et al., 1991). Furthermore, at the protein level, TTR is the major protein synthesized and secreted by the choroid plexus of eutherians, marsupials, monotremes, birds, and reptiles (Harms et al., 1991). The common ancestor of those animals whose choroid plexus specializes in TTR synthesis and secretion are the stem-reptiles, whose brains showed the first traces of neocortex (Kent, 1987). As THs are lipophilic, the increase in lipid volume of the brain could have been a selection pressure in turning on TTR synthesis in the choroid plexus and secreting it into the CSF, to act as a TH-binding protein in the CSF, thereby reducing the partitioning of THs into the lipid membranes and allowing a greater distribution of TH throughout the CSF (Schreiber and Richardson, 1997). Thus, the onset of TTR synthesis in the choroid plexus is suggested to have occurred at the stage of the stem-reptiles, about 300 million years ago (Schreiber and Richardson, 1997). TTR is synthesized in the liver of amphibians and fish during specific stages of development (see Richardson, 2007), but until now synthesis of TTR in the choroid plexus of amphibians and fish has not been described [although TTR mRNA has been detected in whole brain of sea bream (Santos and Power, 1999)]. The main protein synthesized and secreted by amphibian choroid plexus is a lipocalin (Achen et al., 1992), which could be the functional precursor of choroid plexus-derived TTR, as lipocalins are known to bind small hydrophobic molecules. That the choroid plexus specializes in the synthesis and secretion of TTR in such a great range of vertebrates is also suggestive of an important function. Furthermore, the amino acid sequence of TTRs across all vertebrates is so highly conserved, that it forms a single motif (Hennebry et al., 2006). Such high conservation of primary structure can be explained by extremely strong selection pressures during the past 500 million years.

## Altricial vs. precocial brain development and TTR synthesis by the choroid plexus

The brain develops at different rates in different vertebrate species. At the time of birth, the brain of precocial animals (e.g., chickens, sheep) is significantly further developed than the brains of altricial animals (e.g., rats, mice). Therefore, it is of interest to compare the ontogeny of TTR synthesis in the choroid plexus of altricial animals compared with precocial animals.

In rats (23 days gestation), TTR mRNA was first detected in the choroid plexus at E12.5 in the 4th ventricle, at E13.5 in the lateral ventricles, and at E17.5 in the third ventricle. The proportion of TTR mRNA in total RNA increased 40-fold from E12.5 until birth (Fung et al., 1988). Because the choroid plexus develops faster than many other parts of the brain (Sturrock, 1979), part of this 40-fold increase in TTR mRNA could have been due to the increase in size of the choroid plexus with respect to the size of the brain. The peak of TTR mRNA as a proportion of total brain mRNA (about 140% the level in adults) occurred 2 days before birth, just prior to the period of fastest brain growth. By 8 days after birth, TTR mRNA levels had decreased to adult levels (Fung et al., 1988).

In sheep (155 days gestation), TTR mRNA was detected in choroid plexus from very early embryos (Tu et al., 1990). In sheep, the maximum increase in size of the choroid plexus (E70) occurred before that of the rest of the brain (E105). At E40 the proportion of TTR mRNA in total mRNA was 34% of that in adult choroid plexus. By E90, this had increased to 70% of the adult value, and stayed constant at this level during the remainder of gestation (Tu et al., 1990).

In sheep (precocial), the maximal increase in brain weight occurred 50 days *before* birth (Tu et al., 1990), whereas in rats (altricial), the maximal increase in brain weight was about 9 days *after* birth (Fung et al., 1988). The ratio of choroid plexus weight to total brain weight in sheep decreased to a stable value about 20 days *before* birth (Tu et al., 1990), whereas that for rats occurred about 15 days *after* birth (Fung et al., 1988). For both species, the choroid plexus had its maximal growth rate prior to that of the rest of the brain.

The critical period of brain development which is dependent on THs is earlier for precocial animals than for altricial animals (Fisher and Polk, 1989). The maximum TTR mRNA level in the choroid plexus of sheep (precocial) occurred during the first half of gestation, whereas in rats (altricial), it occurred only 2 days prior to birth. If TTR is involved in moving THs across the choroid plexus into the brain, and if this TTR then has a role in TH distribution within the CSF, the timing of the maximal synthesis of TTR by the choroid plexus is highly appropriate.

In summary, in the 1990s it was proposed that T4 bound to a TH distributor protein in blood, is in equilibrium with free T4 which is lipophilic, so partitions into the choroid plexus. Within the choroid plexus, the newly synthesized TTR by the epithelial cells binds to the T4 that entered the choroid plexus from the blood. Choroid plexus-derived TTR is 100% secreted into the CSF (not into the blood), so the TTR-T4 complex would be secreted into the CSF, followed by its distribution via the CSF to the brain tissue. Thus, the TTR synthesized by the choroid plexus is involved in moving T4 from the blood into the CSF, against the free T4 gradient (Chanoine et al., 1992; Southwell et al., 1993; Schreiber and Richardson, 1997). We now know some of these concepts to be outdated (see below).

## Humans lacking TTR have not been reported

Cases of humans lacking either of the other two TH distributor proteins: albumin (Minchiotti et al., 2013) or thyroxine-binding globulin (Refetoff, 1989) have been described. Such people are apparently healthy and without overt clinical symptoms. However, no humans lacking TTR have been reported. In the 1990s the hypothesis was that because TTR is the only TH distributor protein synthesized in the brain, is secreted unidirectionally across the apical surface of the choroid plexus epithelial cells into the CSF, its role in the movement of TH from the blood into the CSF, its intermediate affinity for T4 (between those of albumin and thyroxine-binding globulin), and its role is being the most effective at distributing T4 to cells (see above), that it was thought to be crucial for survival, most probably due to the requirement of adequate amounts of TH for normal CNS development (Harms et al., 1991).

There are cases of humans with mutations in TTR resulting in higher affinity for THs, including Gly6Ser (~12% of the population), Thr119Met, Ala109Thr, and Ala109Val (Refetoff et al., 1996 and references therein). Of these four variants, only Ala109Thr resulted in affected individuals consistently having elevated serum T4 levels (Moses et al., 1982) [although Ala109Val individuals had elevated rT3 levels (Refetoff et al., 1996)]. More than 100 point mutations in human TTR result in TTR amyloidosis (familial amyloidotic polyneuropathy) (Benson, 2009). Whilst the variant TTRs can have differing affinities for T4 (Rosen et al., 1993) the patients are generally euthyroid (no publications indicating otherwise; and personal communication from Merrill Benson). This is possibly because in the blood, only about 1 in 200 TTRs have a TH bound. Therefore, if some of this TTR is deposited as amyloid then it might not affect overall TH distribution. The point being made here is that whilst there are diseases associated with TTR, there are no humans without TTR, whereas there are healthy humans without either albumin or thyroxine-binding globulin.

However, the function of TTR synthesized by the choroid plexus has been debated. Some believe it to be involved with the movement of T4 from the blood into the CSF (based on the functional, developmental and evolutionary data presented above), whilst others dispute this (read below). The controversy arose when TTR null mice were created and were essentially without an overt phenotype.

## TTR null mice

The production of TTR null mice (Episkopou et al., 1993) that were viable and fertile threw the above hypothesis on the role of choroid plexus-derived TTR into debate. TTR null mice had reduced levels of total T4, total T3, retinol, and retinol-binding protein in their blood, but did not show any overt phenotype. Consequently, some concluded that TTR was not important for THs to reach the brain e.g., (Palha et al., 1994, 1997, 2000). One study concluded that there were no differences in the distribution of the THs to tissues of TTR null mice compared with wildtype mice, except in the choroid plexus, where the T4 level was 14% in the TTR null mice compared to wild-type mice (Palha et al., 2000). A semi-quantitative morphological study suggested that wildtype and TTR null mice that had been injected intravenously with ^125^I-T4 or ^125^I-T3 did not show any difference in TH distribution throughout their brains (Palha et al., 2002). This bolstered the interpretation that TTR is not an important TH distributor and that TTR null mice should be considered euthyroid (Palha et al., 1994, 2000; Palha, 2002). However, this did not appear to correspond to the earlier data showing that choroid plexus-derived TTR was involved with movement of T4 from the blood into the brain (Dickson et al., 1987; Schreiber et al., 1990; Chanoine et al., 1992; Southwell et al., 1993).

Soon thereafter, the regulation of neural stem cell cycle in the subventricular zone of adult rodent brain was shown to be dependent on TRα/TH by influencing both cell proliferation and apoptosis (Lemkine et al., 2005). Consequently, it was hypothesized that if TTR was involved in the delivery of THs to the neural stem cell niche, then this population of cells in TTR null mice would be affected. Indeed, TTR null mice were shown to have reduced apoptosis of neural stem cells in the subventricular zone compared to wildtype mice. Furthermore, the level of apoptosis was similar to that of hypothyroid wildtype mice, implying a significant reduction of TH delivery to neural stem cells in this niche (Richardson et al., 2007). It was suggested that because the dissociation rate of T4 from albumin differs so significantly to that of T4 from TTR, and because the concentration of albumin in the CSF is significantly lower than that in the blood, that in the absence of TTR, albumin would be unlikely to distribute significant amounts of TH beyond the ependymal layer of the brain, resulting in the reduction of TH reaching the subventricular zone dur to a shallower TH gradient (Richardson et al., 2007). This study highlighted the importance of TTR in delivering THs to at least one of the two populations of neural stem cells in adult mammalian brain.

Until then, all studies involving TTR null mice had been focussed on adult mice. However, it is well known that THs affect growth and development, particularly of the CNS (Morreale de Escobar et al., 1987). In severe cases, TH deficiency in human babies during gestation results in stunted growth and mental retardation. Therefore, several aspects of growth known to be influenced by TH were examined during post-natal development in TTR null mice. The absence of TTR resulted in delays in several TH-regulated events including bone growth, CNS maturation, intestine and muscle development and in weaning (Monk et al., 2013). Furthermore, there was no specific accumulation of ^125^I-T4 in the choroid plexus of adult TTR null mice, following intravenous injection. This was in stark contrast to the accumulation of ^125^I-T4 in the choroid plexus of adult wildtype mice, following intravenous injection (Monk, 2006). This delay in TH-regulated events in the CNS of TTR null mice indicated that TTR has a role in TH delivery in the CNS during development.

In summary, there was strong evolutionary and developmental evidence that choroid plexus-derived TTR was important for moving THs into the brain, but this was not backed-up by analyses of TTR null mice. Therefore, there had to be another mechanism for THs to enter the brain and presumably the choroid plexus.

## TH transmembrane transporters

The first publication on TH transport that indicated an energy-dependent process for TH uptake into cells was in 1954 (Christensen et al., 1954) but was “lost” in the literature for decades. In the 1970s Rao demonstrated that TH entry into cells was temperature-dependent, saturable, high-affinity, and low-capacity, ligand specific, could transport against a gradient and required metabolic energy (Rao, 1981). Yet some researchers missed these publications and believed that because THs could avidly partition into cell membranes, they could diffuse across the membrane as a mode of entry into cells. For a history of hypotheses about TH entry into cells, see (Hennemann et al., 2001). Currently, it is known that THs enter cells via TH transmembrane transporters, which participate in the influx and efflux of THs into and out of cells. Several classes of TH transmembrane transporter proteins that belong to the family of solute carrier (Slc) transporters have been identified e.g. organic anion transporting polypeptides (OATPs), monocarboxylate transporters (MCTs) and L-type amino acid transporters (LATs), and actively participate in entry and exit of THs into and out of cells (Visser et al., 2011).

Several TH transmembrane transporters have been localized to the choroid plexus (Mayerl et al., 2012) and their distribution in the choroid plexus differs between species (Wirth et al., 2014). Most TH transmembrane transporters identified to date are promiscuous with respect to the range of metabolites they transport. Only MCT8 has been identified as being specific to transport of THs and their derivatives (Kinne et al., 2010). Intriguingly, there exists dramatic species-specificity in TH transmembrane transporters deficiency phenotypes. For example, human males with MCT8 mutations present with mental retardation (IQ <40), speech deficits and severe neurological abnormalities (known as the Allan-Herndon-Dudley syndrome), whereas MCT8 null mice are without an overt neurological phenotype (Kersseboom and Visser, 2011). The discovery of the suite of TH transmembrane transporters, particularly in the choroid plexus, was important in understanding how TTR null mice were without an overt phenotype. The difference in phenotype between MCT8 variant human males and MCT8 null mice, could point toward elucidating why TTR null mice are viable, yet no human lacking TTR has been reported. Possibly, this could be due to different combinations of TH transmembrane transporters in the choroid plexus and at the blood-brain barrier, thereby partially compensating for the lack of choroid plexus-derived TTR in mice (but equivalent mechanisms lacking in humans). For example, Lat2 is synthesized in the choroid plexus of mice but not in the choroid plexus of humans (Wirth et al., 2009) or chickens (Darras et al., 2015). This may be an explanation for the mild phenotype of TTR null mice, yet the apparent absence of humans lacking TTR. For a more detailed discussion on the role of TH transmembrane transporters, deiodinases and the establishment of TH gradients across membranes, see a comprehensive review by Schweizer and Kohrle (2013).

The majority of current information on TH transmembrane transporters has been derived from only human and rodent data. A recent, comprehensive review on expression of TH transmembrane transporters during development in zebrafish, *Xenopus tropicalis*, chicken, and mouse (Darras et al., 2015) highlights how much is still unknown. Whilst the developing choroid plexus has only been analyzed for TH transmembrane transporters in a few species, interesting differences are emerging. For example, zebrafish choroid plexus does not synthesize MCT8, which contrasts with MCT8 synthesis in the choroid plexus of chickens and rodents. Furthermore, Lat2 is synthesized in the choroid plexus of mice but not humans (Wirth et al., 2009) and appears to be totally absent in chickens (Darras et al., 2015).

From an evolutionary perspective, we need to elucidate the developmental expression profiles for each of the TH transmembrane transporters in the choroid plexus from a variety of vertebrates representing each of the vertebrate classes. In particular, a comparison of expression of TH transmembrane transporters in the choroid plexus of animals who synthesize TTR in their choroid plexus (reptiles, birds, and mammals) with those who do not synthesize TTR in their choroid plexus (fish and amphibians) should be made. Furthermore, comparisons of TH transmembrane transporter expression profiles in the choroid plexus between precocial and altricial species would be intriguing. A comparison of the expression profiles of TH transmembrane transporters in mammalian choroid plexus vs. those in choroid plexus from non- mammals should be made, as mammalian TTRs are the exception in having higher affinity for T4 than T3. TTRs from all other vertebrates have higher affinity for T3 than for T4 (see below). Such studies could shed light on the relative roles of each of the TH transmembrane transporters and choroid plexus-derived TTR in the overall movement of THs from the blood into the CSF during development.

## Mammalian TTRs are the exception

In all studied species of fish, amphibians, reptiles and birds, TTR has higher affinity for T3 than T4, whereas in mammals TTR has higher affinity for T4 than for T3 (Richardson, 2007). Thus, mammals are the exception in this regard. Is it possible that whilst TTR made by the choroid plexus of mammals moves T4 into the brain, TTR from other animals moves T3 into the brain? What could have been the selection pressure for the change to TTR from distributing T3 to distributing T4? One possibility could be a greater level of control, requiring an additional level of regulation (i.e., deiodination of T4 to T3) at the target site. For example, different regions of the brain generate different proportions of T3 by local deiodination (van Doorn et al., 1985). Two features that distinguish mammalian brains from non-mammalian brains that are regulated (at least in part) by thyroid hormones are the corpus callosum which is heavily myelinated, and the highly developed cortex. The point here is that mammals may be the exception rather than the rule, when it comes to modes of TH entry into the CNS, so we should not just consider mammalian data. Furthermore, we have seen examples between eutherian species, of significant differences in TH transmembrane transporters (e.g., Lat2).

The literature is often skewed toward data on mammals, especially humans (due to our self-centerd interests in medicine/disease) and mice (due to the ease of working with them: inbred strains, short generation times, knock-in/out genetic manipulations etc), so we should be careful in analysing data from humans and rodents or eutherian mammals and drawing generalized conclusions, which may not be applicable to all mammals, let alone to all vertebrates. Furthermore, data from knock-out mice can sometimes raise more questions than they answer.

## Confusion arising from mouse KO models of TH movement into the brain

TTR null mice have 36% T4 levels in their brain compared to wildtype mice (Palha et al., 1997). This implies that TTR is responsible for the other 64% of T4 moving into the brain. However, Mct8/Oatp1c1 double knock-out mice are cited as having only 10% TH concentration in their brains compared to wildtype mice (Muller and Heuer, 2014). However, careful analysis of the original paper reveals that only the forebrain (and not the entire brain) was analyzed for TH content (Mayerl et al., 2014). Furthermore, if TH transmembrane transporters are responsible for moving significant amounts of TH into and out of the choroid plexus, then why do TTR null mice have only 14% T4 in their choroid plexus compared with wildtype mice? How do these data from TH transmembrane transporter and TTR null mice correlate with the data from Chanoine et al. (1992)? The quantitative contributions of the various mechanisms for TH moving across the blood-CSF barrier (the choroid plexus) and across the blood-brain barrier need to be investigated in greater detail.

## Current hypothesis for TH movement from blood via the choroid plexus to the CSF

**Figure 1:**
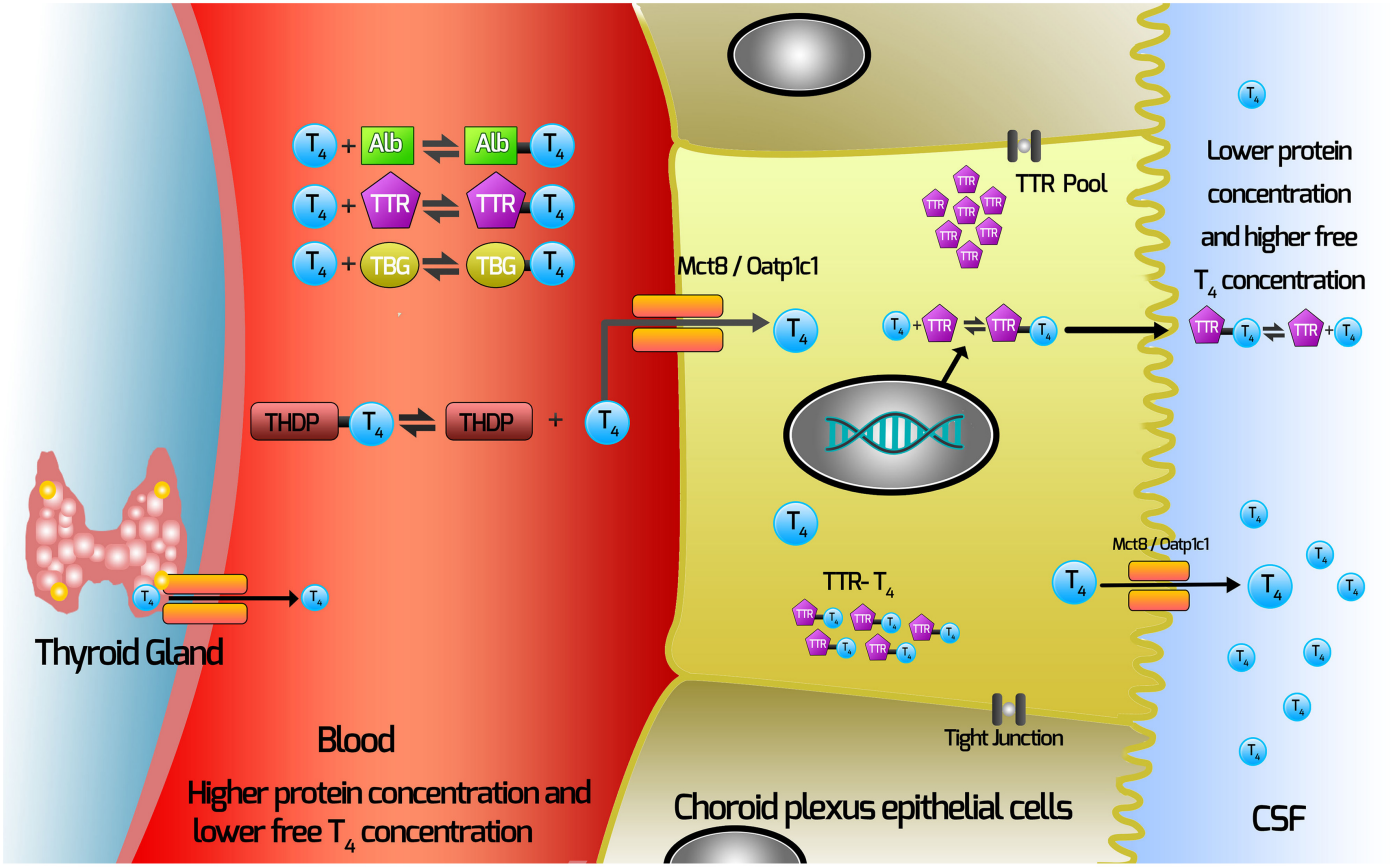
**Current hypothesis of mechanisms involved with TH movement from the blood across the choroid plexus into the CSF**. THs are synthesized in the thyroid gland and secreted into the blood, where >99% are bound by the TH distributor proteins (THDPs) which include albumin (Alb), transthyretin (TTR), thyroxine-binding globulin (TBG) and to a lesser extent by specific lipoproteins, depending on the species and the stage of development. The main form of TH released from the thyroid gland is T4, so T4 is used as the example in this schematic. T4 dissociates from TH distributor proteins in the blood and enters the choroid plexus via TH transmembrane transporters (which may include Mct8 and Oatp1c1, depending on the species). TTR is synthesized by the choroid plexus epithelial cells and is secreted into the CSF. Some T4 binds TTR synthesized in the choroid plexus epithelial cells. T4 moves out of the choroid plexus into the CSF via either secretion of the T4-TTR complex or via TH transmembrane transporters.

Our current hypothesis for the movement of TH from the blood via the choroid plexus is depicted in Figure 1. In this Figure, T4 (major form of TH secreted by the thyroid gland) is depicted.

1. T4 dissociates from TH distributor proteins in the blood.
2. T4 enters the choroid plexus via TH transporters.
3. TTR is synthesized by the choroid plexus epithelial cells.
4. Some T4 will bind TTR synthesized in the choroid plexus epithelial cells.
5. T4 moves out of the choroid plexus into the CSF via either secretion of the T4-TTR complex; or via TH transmembrane transporters.

It is worth noting that there is a higher concentration free T4 in CSF than in blood, so net movement of TH from the blood to the CSF is against this gradient. Therefore, it would not be surprising if several mechanisms for moving TH from the blood via the choroid plexus (and the blood-brain barrier) had evolved.

## Conclusion

From the above controversy regarding the importance of TTR synthesis by the choroid plexus, several points support the hypothesis that choroid plexus-derived TTR plays a role in the movement of THs from the blood to the brain and in distributing THs in the blood and CSF. These points include evolutionary, developmental, molecular and functional data. Therefore, it would follow that TTR synthesized by the choroid plexus would appear to have an important regulatory role in determining the delivery of thyroid hormones into the CSF.

In conclusion, there is evidence for two mechanisms for THs to move out of the choroid plexus into the CSF: (i) secretion of TH bound to choroid plexus-derived TTR, and (ii) efflux of THs via TH transmembrane transporters. The relative quantitative contribution of each of these mechanisms during each stage of CNS development is currently unknown and should be investigated. Furthermore, perhaps the role played by choroid plexus-derived TTR is more significant in non-mammalian vertebrates than in mammals. It would be interesting to analyze the developmental profiles of TH transmembrane transporters in the choroid plexus (and in other blood-brain barrier structures) in non-mammalian vertebrates during development. The delivery of the requisite concentrations of THs to specific areas of the brain at each stage of CNS development is so important, that it would be understandable for evolution to have positively selected for more than one strategy for THs to enter the CNS.

### Conflict of interest statement

The authors declare that the research was conducted in the absence of any commercial or financial relationships that could be construed as a potential conflict of interest.
